# The effect of injection using narrow‐bore needles on mammalian cells: administration and formulation considerations for cell therapies

**DOI:** 10.1111/jphp.12362

**Published:** 2015-01-26

**Authors:** Mahetab H. Amer, Lisa J. White, Kevin M. Shakesheff

**Affiliations:** ^1^ Wolfson Centre for Stem CellsTissue Engineering and Modelling (STEM)School of PharmacyUniversity of NottinghamNottinghamUK

**Keywords:** apoptosis, cell injection, cell therapies, NIH 3T3, viability

## Abstract

**Objectives:**

This study focuses on the effect of the injection administration process on a range of cell characteristics.

**Methods:**

Effects of different ejection rates, needle sizes and cell suspension densities were assessed in terms of viability, membrane integrity, apoptosis and senescence of NIH 3T3 fibroblasts. For ratiometric measurements, a multiplex assay was used to verify cell viability, cytotoxicity and apoptosis independent of cell number. Co‐delivery with alginate hydrogels and viscosity‐modifying excipients was also assessed.

**Key findings:**

Ejections at 150 μl/min resulted in the highest percentage of dose being delivered as viable cells among ejection rates tested. The difference in proportions of apoptotic cells became apparent 48 h after ejection, with proportions being higher in samples ejected at slower rates. Co‐delivery with alginate hydrogels demonstrated a protective action on the cell payload.

**Conclusions:**

This study demonstrates the importance of careful consideration of administration protocols required for successful delivery of cell suspensions, according to their nature and cellular responses post‐ejection.

## Introduction

The success of cell therapies relies on the effective and reliable delivery of viable cells to the target site, where they can produce the desired therapeutic effect. Cell therapy holds promise in the treatment of various diseases, whereby injured tissues are provided with a population of cells that can use cues from its microenvironment to restore function. Preclinical animal studies on cell therapy have been translated into clinical trials for a multitude of central nervous system (CNS) disorders, including Parkinson's disease,[1, 2] stroke[3, 4, 5, 6, 7] and cardiovascular repair.[8] Cell therapies are also attracting attention as potential treatments for irreversible retinal pathologies.[9] However, the clinical translation of cellular therapeutics is hindered by substantial loss of transplanted cells following delivery.[10]

Numerous cell therapy procedures use injection‐based administration to deliver high‐density cell preparations to the target site, either systemically or directly.[11] There are numerous challenges to the delivery of the delicate cell‐based therapeutics, especially those requiring micro‐volumes of cell suspensions to be delivered with high accuracy, such as in the treatment of retinal diseases. Because many cell therapy procedures require the use of syringe‐based devices to deliver cells, particularly in the case of small defects and sites of limited accessibility, there is an urgent need for the development of delivery systems to provide improved cell viability and function. Although physicians may prefer the use of small‐gauge needles, these may affect the viability of cells during their passage through the needle. Research has focussed on the effect of the host microenvironment after injection as a reason for low injected cell viability.[12] However, cell damage may first occur during ejection because of the mechanical disruption of cells. When flowing through a needle, cells may experience various types of mechanical forces, including extensional forces and shear forces attributable to linear shear flow. Forces at the transition point between syringe and needle can also damage cells. In a syringe‐based injection system, the inner diameter of a syringe is typically larger than that of the needle, so cells experience an increase in linear velocity as they pass into the needle. This generates an extensional force that has been suggested to be the main contributor to cell injury during injection. Furthermore, cells and liquid in the middle of a cannula travel at a different flow velocity than those at the walls of the needle, exposing cells to shear stress.[13] With the growing number of clinical trials studying the applicability of cell therapy procedures, a detailed understanding of the limitations of cell delivery is vital. Currently, injection protocols mainly rely on the operator's personal experience through trial and error.[14] Therefore, robust cell delivery systems must be developed and standardised to expedite the translation of cell therapies to the clinic.

To facilitate the translation of cell therapy from bench to bedside, numerous studies have been carried out to assess the effects of the injection process on cell functionality during and after cell delivery. However, a range of needle sizes have been used in injectability studies, complicating comparisons.[13, 15, 16, 17, 18, 19] This has led to conflicting results, with studies showing cell manipulation through a needle affected cell viability,[17] and others stating that flow through a needle did not have a significant effect.[19] However, it would be misleading to assume that the same effects of shear stress occurred on all cells used in cellular therapy due to differences in the size and shape of different cell types. In addition, different investigators had disparate definitions of successful cell transplantation. In an early clinical study on stroke patients, Kondziolka *et al*.[20] found the decrease in viability of neuronal cells after injection by almost 50% clinically acceptable. Investigating viability in cell therapy is crucial because a small level of cell death within a concentrated cell population may have a significant effect on the remaining viable portion by the release of cytotoxic agents.[21]

The aim of this study was to investigate the different aspects of successful delivery of NIH 3T3 cells following ejection from clinically relevant, narrow‐bore needles. This cell line has been widely used as a standard model system for testing wound healing activity *in vitro* because the cells deposit extracellular matrix (ECM) and fill spaces within tissues.[22, 23] The main advantage of this cell line is that a large supply of cells can be obtained with high reproducibility.[24] NIH 3T3 cells have also been frequently used in studies of cell functions such as cell adhesion, movement and proliferation.[25] With a growing number of clinical trials exploring potential applications of cell therapy, understanding the factors that may impact the viability and functionality of these cells post‐injection is of considerable importance. Reviewing current literature, there is a lack of comprehensive testing of the various parameters of cell health and functionality following their ejection. Our use of a comprehensive set of tools for the assessment of cell delivery allows clinicians to make informed judgements regarding the most suitable administration and formulation requirements for cell therapy clinical trials, and answers critical questions regarding possible reasons for failure to deliver sufficient numbers of viable cells.

## Materials and Methods

Materials were obtained from Sigma‐Aldrich (Poole, UK) unless otherwise stated.

### Cell culture

Swiss mouse embryonic fibroblast cell lines (NIH 3T3) were cultured in Dulbecco's Modified Eagle's Medium (DMEM) media (Gibco Life Technologies, Paisley, UK) supplemented with 10% (*v/v*) fetal calf serum (FCS), 1% (*v/v*) penicillin‐streptomycin and 1% L‐glutamine. For routine passaging, a standard trypsinisation protocol was carried out using 0.25% (*w/v*) trypsin/2 mM EDTA solution. In Annexin V/PI analyses, cells were detached using Accutase Cell Dissociation Reagent. Cells used were between passages 29–41.

### Standard cell preparation protocol for experimental syringe manipulation procedures

After trypsinisation, cells were centrifuged at 180 × *g* for 5 min, and then reconstituted to a cell density of 5 × 10^5^ cells/ml in phosphate buffered saline (PBS), unless otherwise stated. Cell doses in this study were selected conservatively on the basis of previous clinical studies[26, 27, 28, 29] and the rapid growth characteristics of the cells. There were 100 μl of aliquots of this final concentration used for injection experiments. Cells were directly pipetted to provide a control. For cell manipulation, 100 μl of Hamilton Hamilton Gastight® syringes (GASTIGHT) syringes (model 1710RN), fitted with standard and customised removable needle (RN) stainless steel needles were used (Hamilton, Bonaduz, Switzerland). Cell suspensions were drawn up using a Harvard Infuse/Withdraw syringe pump (Model PHD 2000, Harvard Apparatus, MA, USA) at a constant rate of 300 μl/min before being ejected at various controlled rates into 1 ml of complete media. Needle sizes were chosen to be relevant to high accuracy cell therapy applications. Reviewing the literature, ejection rates used in clinical trials are highly variable: For neural cell transplantation for example, some using a rate as low as 5 ul/min,[30] some ranged between 10–1000 μl/min for stroke, and about 6 ml/min for Parkinson's disease.[7, 31] Ejection rates were chosen to mimic clinically relevant ejection rates while still being feasible to use with a syringe pump, to provide accurate control over ejection rates.

### Trypan blue exclusion method

After ejection, trypan blue (Fisher Scientific, Loughborough, UK) was added to 10 μl of the cell suspension in a ratio of 1:1 and mixed gently, then counted using the improved Neubauer haemocytometer (Scientific Laboratory supplies, UK).

### PrestoBlue assay

PrestoBlue (Invitrogen Life Sciences, Paisley, UK) was used to measure 6‐h and 24‐h viability post‐injection as well as proliferation over several days. One microlitre of a 1:9 mixture of PrestoBlue: culture medium was added to each well, and incubated at 37°C for 45 min in the dark. Triplicate 100 μl of aliquots from each well were measured on a Tecan Infinite M200 microplate reader (Tecan, Reading, UK) using excitation and emission wavelengths (Exc/Em) of 560/590 nm.

### Live/Dead viability/cytotoxicity assay

Assessment of cell viability was performed according to the manufacturer's instructions (Invitrogen Life Technologies, Paisley, UK). Calcein AM and ethidium homodimer‐1 (EthD‐1) were prepared in PBS to produce the Live/Dead staining solution. Samples were visualised using fluorescence microscopy (Leica Microsystems Ltd., Milton Keynes, UK), where live cells stained green and dead cells stained red.

### Flow cytometry analysis

Cell suspensions were ejected into Eppendorf tubes to ensure that no cell suspension was lost during ejection. These were then immediately transferred to flow cytometry tubes and analysed. Cell suspensions (5 × 10^6^ cells/ml of PBS) were analysed using a Beckman Coulter Cytomics FC500 flow cytometer (High Wycombe, UK) using a 488 nm laser. For Live/Dead analysis, a sorting parameter of 50,000 total events was used per sample, or 300 s. For Annexin V/PI, a sorting parameter of 30,000 total events was used. Data were analysed using WEASEL software (F. Battye, Walter and Eliza Hall Institute, Melbourne, Vic., Australia). Quadrants were determined using unstained and single stain control samples.

In Live/Dead analysis, viability was determined by dividing the number of viable events (events fluorescing in the lower right quadrant) by total number of events that occurred within the control. Using this method allows the number of cells that may have lysed, and therefore not produced an event, to be taken into account.

For the detection of apoptosis, cells were analysed using the Alexa Fluor 488 Annexin V/Dead Cell Apoptosis Kit (Molecular Probes, UK). The method used was loosely based on the protocol described by Rieger *et al*.[32] Briefly, cells were detached using Accutase after the desired period of incubation post‐injection, washed with PBS and centrifuged at 335 × *g* for 8 min. Cells were re‐suspended in 100 μl of 1X Annexin V‐binding buffer, then 5 μl of Annexin V‐FITC was added and incubated for 10 min. Afterwards, 1 μl of propidium iodide (PI) was added and incubated for 15 min. Annexin‐binding buffer was then added, and stained cells were kept on ice in the dark until analysis. Cells treated with 3 μM staurosporine were used as positive control.

### Senescence assay

Of the cell suspensions, 70 μl was ejected at various rates, cultured in T‐25 tissue culture flasks and incubated for 5 days. Cells were tested for senescence using SA‐β‐galactosidase histochemical staining kit according to the manufacturer's protocol. Senescence‐associated β‐galactosidase (SA‐β‐Gal) activity was quantified by counting the number of stained cells, calculating a final average percentage of the number of stained to total cells. At least 200 cells were counted for each condition from 10 separate microscopic fields in each flask. Hydrogen peroxide (150 μM for 3 h) was used as positive control.

### Apo‐Tox Glo Triplex Assay

Viability, cytotoxicity and apoptosis were measured using the ApoTox‐Glo Triplex Assay (Promega, UK) according to manufacturer's instructions. Cells were cultured at 20,000 cells/well in clear‐bottom 96‐well plates (Costar, Corning Life Sciences, NY, USA). Fluorescence and luminescence readings were made using a Tecan plate reader, and data were normalised to the medium‐only sample. To normalise well‐to‐well variability, cytotoxicity and caspase measurements were represented as normalised to viability measurements (dead/live cell ratio and apoptotic/live cell ratio). Cells subjected to 3 h of 1 μM staurosporine treatment were used as positive control.

### Formulation and preparation of carriers for cell delivery

Alginate gels were prepared in tissue culture water under aseptic conditions, and sterilised by tyndallisation to maintain alginate's properties. Tyndallisation was carried out by heating the prepared gels three times at 70°C for 20 min each at 24‐h intervals. The 1% *w/v* sodium alginate solution (Acros Organics, Geel, Belgium) was mixed using two luer‐locked syringes with sterile‐filtered 0.25% *w/v* calcium chloride solution to cross‐link it. Aqueous cell carriers were prepared using high viscosity carboxymethylcellulose (CMC) (Catalogue number 12M31P, Ashland Speciality Ingredients, Poole, UK) in tissue culture water at a concentration of 0.5% *w/v,* then sterile‐filtered. Cells were suspended in PBS and incorporated into the carriers indirectly via gentle mixing to give a final cell density of 5 × 10^5^ cells/ml.

### Statistical analysis

All analyses were performed using GraphPad Prism 6 software. Data sets were tested for normality and appropriate tests of comparisons were subsequently chosen. For multiple data comparisons, analysis was carried out using the Kruskal–Wallis analysis of variance, unless stated otherwise. Results were considered statistically significant if *P* < 0.05.

## Results

### Effect of varying ejection rates

**Figure 1:**
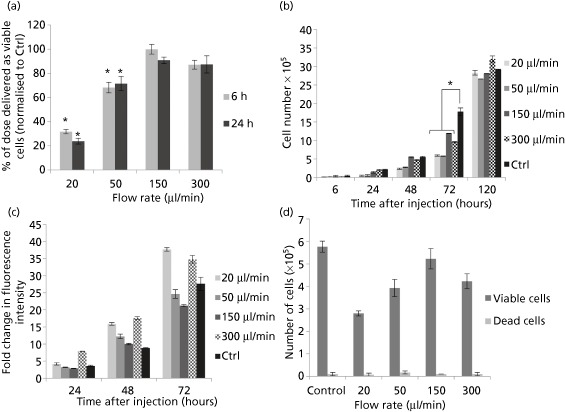
(a) Percentage of NIH 3T3 cells delivered as viable cells, determined using PrestoBlue, following ejection at various flow rates through a 30G needle. Results are mean values ± Standard error of mean (SEM) (*n* = 3). Data were normalised against control value of directly plated cells. Asterisks represent significant difference between samples (*P* < 0.05) and control. (b) Proliferation of NIH 3T3 cells, measured using PrestoBlue, following ejection at various flow rates through a 30G needle. Results are mean values ± SEM (*n* = 3). (c) Proliferation data given as the fold change in mean fluorescence intensity, measured using PrestoBlue, following ejection at various flow rates through a 30G needle from Day 0 of each sample. Results are mean values ± SEM (*n* = 3). (d) Quantitation of cell viability using Trypan blue following ejection of NIH 3T3 cells at various flow rates (mean ± SEM; *n* = 2).

The graph in Figure 1a shows immediate (6 h) and 24‐h cell viability measurements following ejection of cell suspensions at various flow rates measured using the PrestoBlue assay. The percentage of the dose delivered as viable cells was only significantly reduced from the control value at the slower flow rates tested (*P* < 0.05). Cells injected at 150 μl/min showed the highest viability at 99.9% ± 4.07%, with all other ejection rates exhibiting a lower percentage of cells delivered. The proliferative ability of the NIH 3T3 cells was significantly affected following ejection, with all ejection rates showing significantly lower cell numbers than the directly plated control. However, fibroblasts seemed to proliferate normally, with a significant recovery in proliferative ability after 72 h (Figure 1b).

Cell counts, using the trypan blue exclusion method, after injection of cells at different ejection rates are presented in Figure 1c. The percentage of dead cells was only 1.5–2% of the total number of cells in all samples tested. However, the total number ejected was significantly lower at slower ejection rates, suggesting loss of cells during ejection.

All injected samples attached to culture surfaces within 4 h of ejection and showed normal morphology. The Live/Dead staining kit, which relies on esterase activity of living cells (green fluorescence indicates living cells stained by Calcein‐AM) and compromised membranes of dead cells (red fluorescence indicated by EthD‐1), was used to assess viability. Among the ejection rates tested, the percentage of viable cells was high (Figure S1), and a visibly lower number of cells appeared in ejected samples, especially at 20 μl/min.

**Figure 2:**
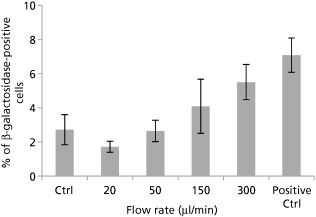
NIH 3T3 cells undergoing senescence following delivery via 30G needles (*n* = 3). Senescence‐associated β‐galactosidase assay was performed at 5 days after injection, with 150 μM H_2_O_2_ for 2 h as positive control. Results are mean values ± SEM.

SA‐β‐Gal was used as a senescence marker to investigate the percentage of cells undergoing senescence post‐injection. Figure 2 shows the quantitation of blue‐stained SA‐β‐gal‐positive cells observed in cells after manipulation, with those injected at 300 μl/min exhibiting a visible increase in β‐galactosidase activity, which was not statistically significant.

### Effect of varying needle gauges and lengths

Finer needles are required for high accuracy applications to minimise injury, especially for neuronal or retinal regenerative applications. To investigate cell viability following injection through different needle sizes, two different experiments were performed. In the first set of experiments, NIH 3T3 cells were injected through 30G, 32G and 34G needles of two lengths, 51 and 20 mm, at 150 μl/min. In the second set, they were injected through 30G and 34G 20‐mm needles at the ejection rates under investigation.

**Figure 3:**
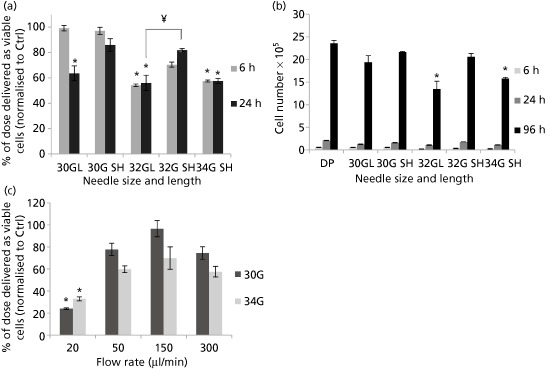
(a) Percentage of NIH 3T3 cells, delivered as viable cells, after ejection at 150 μl/min using various needle gauges and lengths. Results are mean values ± SEM% (*n* = 3). Asterisks represent significant difference between samples and control, and ¥ represent significant difference between needle lengths (Short (SH) = 20 mm; Long (L) = 51 mm) of the same needle gauge, using the Kruskal–Wallis test. (b) Proliferation of NIH 3T3 cells ejected at 150 μl/min using different needle gauges and lengths (mean values ± SEM). Asterisks represent significant difference between samples (*P* < 0.05) and control. DP, directly plated control. (c) Percentage of NIH 3T3 cells, delivered as viable cells, 6 h following ejection at various flow rates using 30G and 34G 20‐mm needles. Results are normalised mean values to control ± SEM (*n* = 3). Asterisks indicate statistically significant differences between control and ejected samples using the Kruskal–Wallis test (*P* < 0.05).

Due to the extremely small diameter of 34G needles, it was impossible to draw up cell suspensions using the 51‐mm needle, and this length was therefore excluded. Figure 3a shows that varying needle gauge and length had a significant effect on the percentage of cells successfully delivered; this was reduced by decreasing internal diameter and increasing needle length. In terms of the cells’ proliferative ability, only the 51‐mm 32G and 20mm 34G needles exhibited significant differences in cell number compared to the control after 96 h of incubation (Figure 3b).

Both 30G and 34G 20‐mm needles displayed the same trend of cell viability with the different ejection rates tested, with cells ejected at 150 μl/min exhibiting the highest viability (Figure 3c). However, the 34G needle showed an evidently larger decrease in the percentage of cell dose delivered than the 30G needle at most ejection rates under investigation (Figure 3c).

### Flow cytometric analysis of NIH 3T3 post‐injection

**Figure 4:**
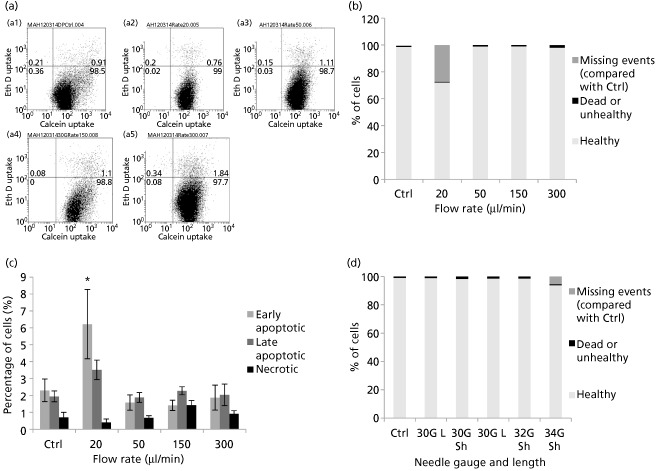
(a) Flow cytometric dot plot quadrant analysis of NIH 3T3 cells, using Live/Dead stain, (a1) directly plated or ejected at (a2) 20, (a3) 50, (a4) 150 or (a5) 300 μl/min. (b) Graph showing results of flow cytometric analysis of NIH 3T3 cells ejected at various flow rates, classified as healthy, dead/unhealthy or missing events (compared with control). (c) Percentages of apoptotic and necrotic cells were analysed by flow cytometric analysis of NIH 3T3 cells 48‐h post‐injection, by double labelling with Alexa Fluor 488 Annexin V and propidium iodide (PI). Mean values of five independent experiments are shown ± SEM. Asterisk represents significant difference between samples and control using two‐way ANOVA followed by Tukey's post‐hoc test. (d) Graph showing results of flow cytometric analysis of NIH 3T3 cells ejected using various needle gauges and lengths, classified as healthy, dead/unhealthy or missing events (compared with control).

Flow cytometry was used as an additional tool for analysis of cellular health. Flow cytometry‐based Live/Dead assay was used for the simultaneous detection of viable and dead cells, giving an indication of both esterase activity and cell membrane integrity. At all ejection rates tested, over 97% of the cells were positive for the cell viability marker calcein‐AM (Figure 4a, lower right quadrant of dot plots), with no major differences in dead cell fractions for different flow rates. This correlates with our previous findings by microscopical examination (Figure S1). However, the sample injected at 20 μl/min displayed a lower total number of events in comparison with all the other tested samples and control (Figure 4b).

Apoptosis was investigated by analysing the percentage of apoptotic cells using Annexin V/PI double staining. This method enables the detection of viable, apoptotic and necrotic cell populations: Annexin V^‐^/PI^‐^ cells were considered viable, Annexin V^+^/PI^‐^ as early apoptotic, Annexin V^+^/PI^+^ as late apoptotic and Annexin V^‐^/PI^+^ necrotic. After 48 h of incubation, differences in percentages between the samples were apparent at the slower ejection rate used (Figure 4c). The proportion of apoptotic cells was higher in the samples injected at slower ejection rates (6.22 ± 2.05% early apoptotic cells at 20 μl/min, versus 1.88 ± 0.74% at 300 μl/min). The results indicate that a significant loss of NIH 3T3 cell viability may still occur 48‐h post‐injection, with the differences in apoptotic cell populations between the injected samples and control being statistically significant at 20 μl/min using one‐way ANOVA, with a Tukey–Kramer multiple comparisons test.

Figure 4d summarises flow cytometric analyses using Live/Dead staining for cell populations ejected at 150 μl/min. There was no visible difference in non‐viable cell numbers with various needle sizes, with viable cells ranging between 98–99.2% of total cells analysed. However, the 34G needle displayed a lower total number of events through the flow cytometer, which was hypothesised to occur due to cell lysis.

### Effect of varying cell density

Cell viability was analysed for cell suspensions of different densities ejected using a 30G needle at two flow rates, to investigate possible effects of cell densities at both low and high ejection rates. With the exception of the highest cell density tested (5 × 10^6^ cells/ml), which showed a higher percentage of dose delivered than other densities under investigation, there were no significant differences in cell delivery between the lower cell densities (Figure S2). Flow cytometric analyses of samples of different cell densities ejected at 150 μl/min through a 30G needle showed no significant differences in viability.

### Investigating cell fate at low ejection rates

Further investigations were required to identify the reason behind the lower number of cells ejected at the slower ejection rates. For a more complete picture, the slower ejection rate of 10 μl/min was also assessed. After ejecting cells, each injection was followed by 3 × 100 μl of PBS at an ejection rate of 300 μl/min to dislodge any cells that may have adhered to the inner surface of the needle. Washes were pooled together, placed in 2‐ml media in a six‐well plate and assessed for viability.

**Figure 5:**
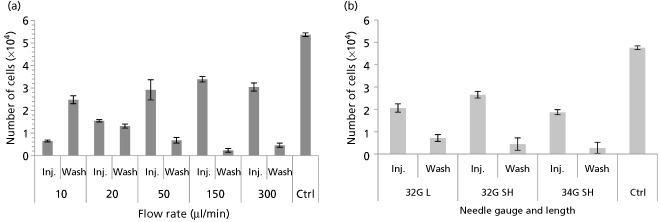
(a) To investigate cell fate at low flow rates, each cell ejection was followed by needle washes (3 × 100 μl of phosphate buffered saline (PBS) ejected at 300 μl/min each), and the number of cells recovered was measured using PrestoBlue (mean ± SEM, *n* = 3). (b) Ejections through needles of various gauges and lengths were followed by washes using 3 × 100 μl of PBS ejected at 300 μl/min each, and the number of cells recovered was measured using PrestoBlue (mean ± SEM).

Figure 5a shows cell numbers after ejection and for the following PBS‐washing step. A trend can be clearly observed, where the number of viable cells in PBS washes increases with slower ejection rates, indicating that the adhesive nature of this fibroblast cell line may be causing them to adhere to needle's inner surface at slower ejection rates. In the case of smaller needle gauges, the number of NIH 3T3 cells in PBS washes was not substantial (Figure 5b), indicating that the lower number of events in 34G flow cytometry analyses was probably due to cell lysis (Figure 4d).

### Assessing compound effects using a multiplex assay

**Figure 6:**
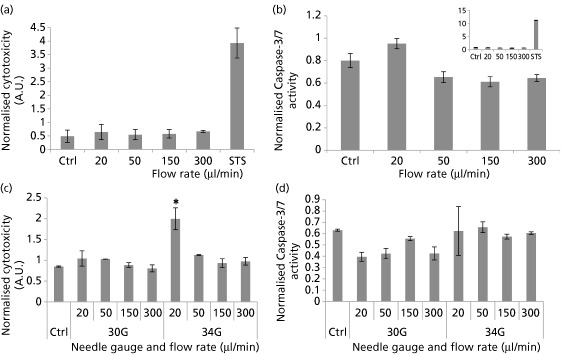
(a) Cytotoxicity in NIH 3T3 cells 4‐h post‐injection (analysed by ApoTox‐Glo Triplex Assay). Cytotoxicity fluorescence at wavelengths 485EXT and 520EM were normalised to viability within the same well. (b) Caspase‐3/7 activity luminescence was represented as normalised to viability in well‐to‐well normalisation. Staurosporine treatment (1 μM) was used as a positive control, and the graph showing its effect is superimposed for clarity (*n* = 3; mean ± SEM). (c) Cytotoxicity in NIH 3T3 cells 4‐h post‐injection (analysed by ApoTox‐Glo Triplex Assay). Cytotoxicity readings were normalised to viability within the same well. (d) Caspase‐3/7 activity luminescence was represented as normalised to viability measurements. Asterisks represent significant difference between sample and control (*P* < 0.05). (*n* = 2; mean ± SEM).

Multiplex assays are capable of measuring multiple parameters (cell viability, cytotoxicity and apoptosis) in a single well independent of cell number. This is desirable to help to provide normalised controls to overcome well‐to‐well variations in cell numbers. Despite the high sensitivity of this assay, there was no statistically significant change in cytotoxicity or caspase‐3/7 activity between the various ejection rates under investigation (Figure 6a and 6b). There were no significant differences in normalised cytotoxicity or caspase‐3/7 activity measurements between samples injected using 30G and 34G 20‐mm needles and the directly pipetted control, with the exception of cytotoxicity in cells injected at 20 μl/min through 34G needles (Figure 6c). A trend toward higher apoptosis occurs with decreasing needle bore size (Figure 6d). However, this fails to attain statistical significance.

### Improving viability during needle flow using viscous carriers

**Figure 7:**
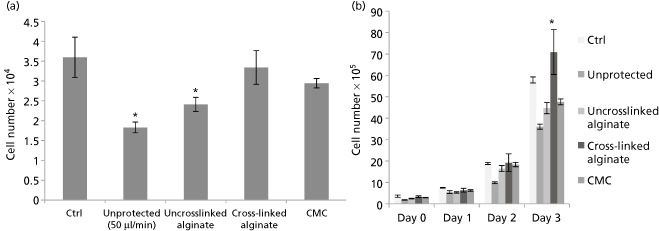
(a) To investigate the proportion of NIH 3T3 cells delivered within phosphate buffered saline (PBS), uncross‐linked 1% alginate solution, 1:4 cross‐linked alginate gels and high viscosity CMC after ejection through a 30G syringe needle at 50 μl/min, normalised to directly plated control (mean ± SEM%, *n* = 3). (b) Cell proliferation of NIH 3T3 cells when injected unprotected in PBS and in several carriers (*n* = 3, mean ± SEM). Asterisks indicate statistically significant improvement in proliferation of ejected cells compared with the directly plated control cells at 72 h (*P* < 0.05); CMC, carboxymethylcellulose.

Because shear stress experienced during the cell injection process may result in a decrease in proportion of viable cells delivered, it was hypothesised that co‐injecting the cells with viscous carriers may improve viability. One per cent of uncross‐linked alginate, cross‐linked (1% *w/v*, 1:4 Ca^2+^: alginate cross‐linking ratio) alginate and high viscosity CMC solutions were used to test this hypothesis. By incorporating the cells in a protective viscous medium, improved cell delivery was demonstrated (Figure 7a). Suspending cells within cross‐linked alginate hydrogels significantly improved the 6‐h viability of ejected cells to 95.6 ± 10.8%, whereas suspending in the viscous CMC carrier improved viability to 85.4 ± 3.03%. In contrast, uncross‐linked alginate did not provide any significant cell protection. The protective effects of the cross‐linked alginate also resulted in an increased proliferation of the cells, with significantly higher cell numbers after 3 days of incubation (Figure 7b).

## Discussion

The treatment potential of cellular therapies depends on the viability and functionality of cells post‐injection. Presently, limited data exist regarding factors vital for the survival of cells during and following ejection via narrow‐bore needles at clinically relevant ejection rates. In this study, we demonstrated the effects of administration stresses on NIH 3T3 cells, a widely used model in cellular biology. This study highlights potential parameters, such as the use of optimum ejection rates, different needle sizes and the nature of injected cells, required to maintain the high viable cell density needed for cell therapy applications.

An overview of assays used to evaluate the various aspects of cellular health post‐injection is shown in Figure S3. The effects of injection delivery on NIH 3T3 cells were quantified in terms of immediate and 24‐h viability, apoptosis and other parameters of cellular health. The PrestoBlue assay measured viability in terms of cell numbers acquired in comparison with the directly pipetted control. Following manipulation within a syringe, the percentage of NIH 3T3 cells delivered as viable cells was not directly proportional to ejection rate as expected, with cells injected at 150 μl/min demonstrating a higher percentage of cells delivered than those injected at other ejection rates. This may be due to striking an optimal balance between the magnitude of shear stress exerted and the time period exposed to mechanical forces within the needle. These viability findings were in agreement with results obtained from counting cells with the trypan blue exclusion test, where slower ejection rates resulted in the ejection of a lower total number of cells. Flow cytometric analyses measured actual proportions of dead cells, which came out of the needle within samples under investigation. Live/Dead analyses demonstrated that cells obtained by injecting through 30G needles at all ejection rates were not discernibly impacted in terms of membrane integrity. The improved proliferation after 72 h of injection may be due to cellular repair mechanisms being activated, which suggests that cell recovery is possible in a favourable environment. In addition, cells may have undergone exponential growth by this time point.

Needle gauge and length also have a significant effect on cell viability post‐injection. A lower percentage of cell dose delivered as viable cells was observed with increasing needle length and decreasing needle diameter. This can be explained by the mechanical forces to which cells are exposed as they flow through the needle: cells are exposed to shear and extensional forces for a longer period of time in longer needles, whereas smaller needle diameters will result in higher force magnitudes.

No significant difference in caspase‐3/7 protein levels, an early regulatory event in apoptosis, occurred upon cell injection. The multiplex assay used confirmed that the ejection rates under investigation did not significantly increase NIH 3T3 cytotoxicity or apoptosis levels at the tested time point. Even upon decreasing needle bore diameter, no significant increase in caspase‐3/7 levels was observed (Figure 6). On 48‐h post‐injection, more visible differences between ejection rates under investigation became apparent with Annexin V/PI staining. Because the lowest ejection rates were translated into a significant increase in apoptotic cell proportions (Figure 4c), this confirms the hypothesis that a balance is needed between the magnitude of mechanical forces exerted on the cells and the time spent exposed to these forces. NIH 3T3 cells are also known to be quite resistant to the induction of apoptosis,[33] which may explain the non‐significant difference in caspase activity despite decreasing needle gauge or increasing ejection rate. Furthermore, senescence was only increased in samples injected at the highest ejection rate (300 μl/min), but this also failed to reach significance (Figure 2).

Because apoptosis and cytotoxicity results did not fully explain the significant differences in the percentages of doses delivered as viable cells, PBS washes were made to investigate the possibility of cells adhering to the inner walls of the needle, since cells used in this work are anchorage‐dependent. Results show that the cells were retained in the dead space of the syringe and on the inner walls of the needle and were only dislodged by PBS washes at a flow rate of 300 μl/min. NIH 3T3 cells adhesive properties are well known and have been studied previously.[34] These results highlight the importance of tailoring cell delivery systems to the nature of the transplanted cells because cells adhering in substantial amounts to the device will compromise the intended therapeutic dose.

It was hypothesised that increasing cell concentration may increase shear forces, resulting in more cell death. On the contrary, increasing NIH 3T3 cell concentration caused a significant improvement in viability at the highest cell density under investigation. It was observed that significant cell clumping occurred at cell densities higher than 5 × 10^6^ cells/ml, which did not allow for reproducible experimentation. The clumping that occurred at this density, although not being substantial enough to cause experimental problems, may have exerted a protective action on the cells. This agrees with previous findings regarding cell aggregation.[35]

Preliminary viability studies carried out on the use of hydrogels and viscosity‐modifying excipients for cellular delivery demonstrated the protective effects of shear‐thinning alginate hydrogels on cells undergoing needle flow. Cross‐linked hydrogels have been reported to undergo plug flow.[36] The hydrogel adjacent to the walls undergoes shear thinning to form a fluid layer that acts as a lubricant. This lubricating fluid layer and plug flow behaviour may be the mechanisms by which an increased proportion of the cells are delivered, reducing the detrimental effects of shear and extensional forces as well as retention of the cells in the delivery device. A similar result was previously obtained by Aguado *et al*. with a 28G needle and 1‐ml syringe.[13] This work confirms that the protective action of co‐delivery with alginate gels is also applicable to adverse effects induced by injection flow in microsyringes and smaller needle sizes. This may be vital to high‐accuracy applications and cells that may display biological changes after exposure to mechanical forces. Such delivery strategies have promising applications for the clinical translation of cell therapy in high accuracy regenerative medicine applications.

## Conclusions

We have demonstrated the effects of manipulation of NIH 3T3 cells using various needle gauge sizes and lengths, cell densities and ejection rates through a needle‐based delivery device. Early loss of injected cells may be due to a combination of cell disruption by mechanical forces exerted during the injection process, cell retention in the needle or the syringe's dead space, and/or cells’ attachment to the delivery system. The higher proportion of apoptotic cells appearing 48‐h post‐injection suggests that the injection process may also induce delayed death through induction of apoptosis. Co‐delivery with viscosity‐modifying excipients demonstrated a protective action on cell payload. Therefore, this study demonstrates the importance of careful consideration of the administration protocol of cell suspensions and the optimisation of delivery parameters according to the nature and cellular responses of cells post‐ejection.

## Declarations

### Conflict of interest

The Authors declare that they have no conflicts of interest to disclose.

### Funding

M.H. Amer is partly funded by a University of Nottingham International Office scholarship and by Misr El‐Kheir Foundation. The project is supported by the UK Regenerative Medicine Platform Hub for Acellular Technologies.

## Supporting information


**Figure S1** Representative fluorescence images showing Live/Dead‐stained NIH‐3T3 cells injected at various flow rates at 48 h of incubation. Live cells exhibited green fluorescence while dead cells showed red fluorescence (Scale bar: 100 μm)
**Figure S2** (a) Comparison of 6‐h viability of different cell densities of NIH‐3T3 cells injected, using a 30G needle, at two different flow rates (20 and 150 μl/min); Results are mean ± SEM% (*n* = 3). Asterisks indicate statistically significant difference between ejected samples and 5 × 10^5^ cells/ml (*P* < 0.05) (b) Flow cytometric dot plot quadrant analysis of NIH‐3T3 cells, using Live/Dead stain, of a cellular density of 5 × 10^5^ (a) 1 × 10^6^ (b) and 5 × 10^6^ (c) cells/ml, ejected at 150 μl/min.
**Figure S3** An overview of assays used in this study to evaluate the various aspects of cellular health post‐injection.
