# Supplementary material for: The effect of injection using narrow‐bore needles on mammalian cells: administration and formulation considerations for cell therapies
Source: J Pharm Pharmacol. 2015 Jan 26;67(5):640–50. doi: 10.1111/jphp.12362 (PMC4964945; doi:10.1111/jphp.12362)

**The Effect of Injection using Narrow Bore Needles on Mammalian Cells: Administration and Formulation Considerations for Cell Therapies**

Mahetab H. Amer, Lisa J. White and Kevin M. Shakesheff

*Correspondence to kevin.shakesheff@nottingham.ac.uk*

## Figure 1S

Representative fluorescence images showing LIVE/DEAD®-stained NIH-3T3 cells injected at various flow rates at 48 hours of incubation. Live cells exhibited green ﬂuorescence while dead cells showed red ﬂuorescence (Scale bar: 100 μm)

| 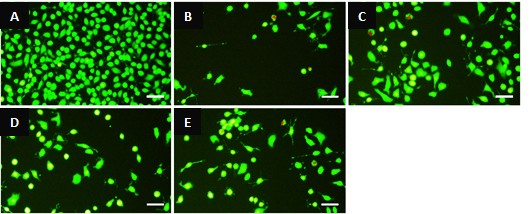  ***50 µL/min***  ***20 µL/min***  ***Ctrl***  ***150 µL/min***  ***300 µL/min*** |
| --- |

## Figure 2S

(A) Comparison of 6 hour viability of different cell densities of NIH-3T3 cells injected, using a 30G needle, at two different flow rates (20 and 150 μL/min); Results are mean ± SEM% (*n*=3). Asterisks indicate statistically signiﬁcant difference between ejected samples and 5x10^5^ cells/mL (*p* < 0.05) (B) Flow cytometric dot plot quadrant analysis of NIH-3T3 cells, using Live/Dead stain, of a cellular density of 5x10^5^ (A) 1x10^6^ (B) and 5x10^6^ (C) cells/mL, ejected at 150 μL/min.

## Figure 3S

An overview of assays used in this study to evaluate the various aspects of cellular health post-injection


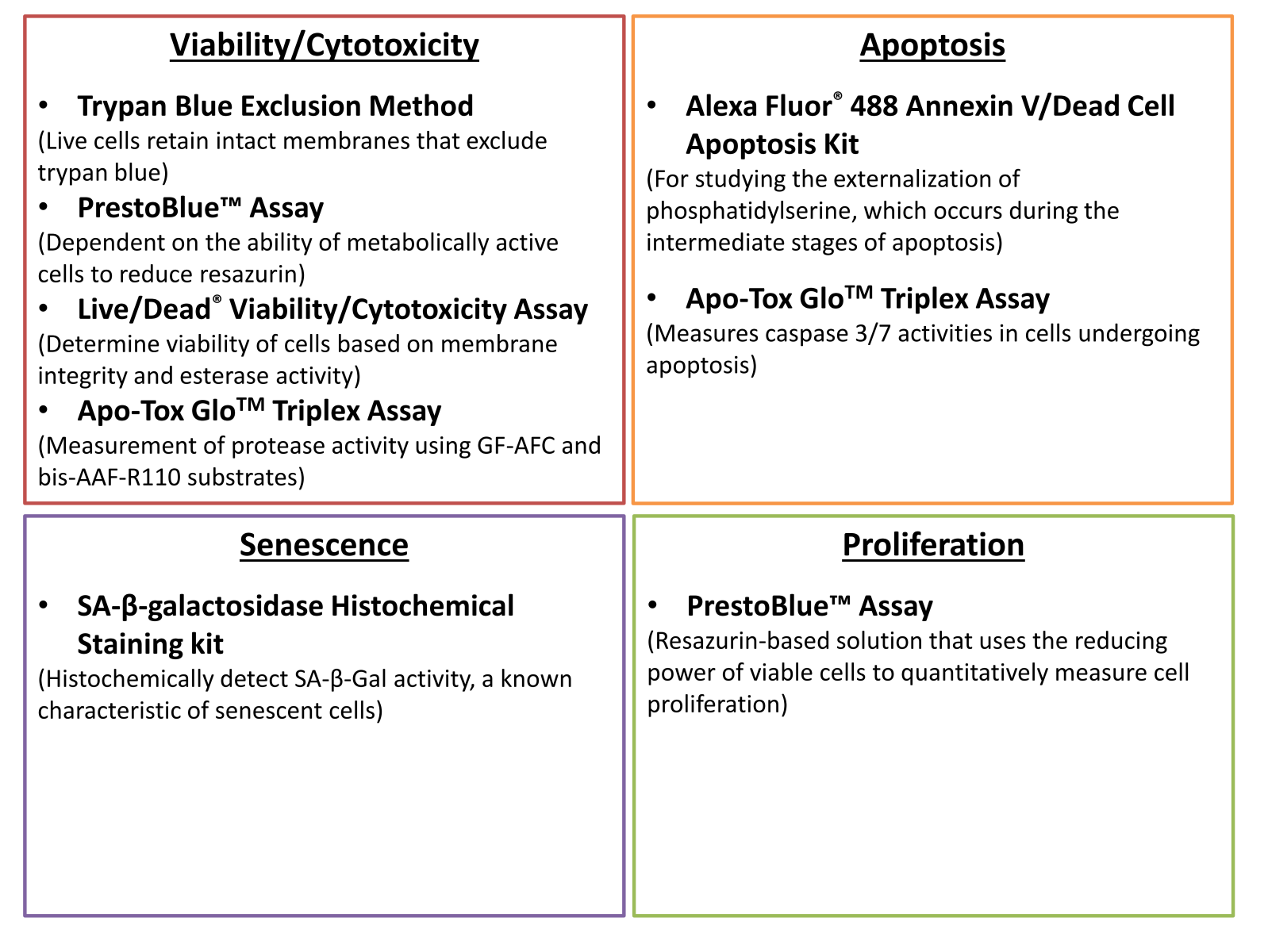

Supplement: Supplementary file 1 — Figure S1 Representative fluorescence images showing Live/Dead‐stained NIH‐3T3 cells injected at various flow rates at 48 h of incubation. Live cells exhibited green fluorescence while dead cells showed red fluorescence (Scale bar: 100 μm) Figure S2 (a) Comparison of 6‐h viability of different cell densities of NIH‐3T3 cells injected, using a 30G needle, at two different flow rates (20 and 150 μl/min); Results are mean ± SEM% (n = 3). Asterisks indicate statistically significant difference between ejected samples and 5 × 10^5^ cells/ml (P < 0.05) (b) Flow cytometric dot plot quadrant analysis of NIH‐3T3 cells, using Live/Dead stain, of a cellular density of 5 × 10^5^ (a) 1 × 10^6^ (b) and 5 × 10^6^ (c) cells/ml, ejected at 150 μl/min. Figure S3 An overview of assays used in this study to evaluate the various aspects of cellular health post‐injection. [file JPHP-67-640-s001.docx]
